# DAQUA-MASS: An ISO 8000-61 Based Data Quality Management Methodology for Sensor Data

**DOI:** 10.3390/s18093105

**Published:** 2018-09-14

**Authors:** Ricardo Perez-Castillo, Ana G. Carretero, Ismael Caballero, Moises Rodriguez, Mario Piattini, Alejandro Mate, Sunho Kim, Dongwoo Lee

**Affiliations:** 1 Information Technologies & Systems Institute (ITSI), University of Castilla-La Mancha, 13071 Ciudad Real, Spain; anaisabel.gomez@uclm.es (A.G.C.); ismael.caballero@uclm.es (I.C.); moises.rodriguez@uclm.es (M.R.); mario.piattini@uclm.es (M.P.); 2 AQC Lab, 13051 Ciudad Real, Spain; 3 Lucentia Lab, University of Alicante, 03690 San Vicente del Raspeig, Alicante, Spain; amate@lucentialab.es; 4 Department of Industrial & Management Engineering, Myongji University, Seoul 449-728, Korea; shk@mju.ac.kr; 5 GTOne, Seoul 07299, Korea; leewow@gtone.co.kr

**Keywords:** data quality, data quality management processes, ISO 8000-61, data quality in sensors, Internet-of-Things, IoT, Smart, Connected Products, SCPs

## Abstract

The Internet-of-Things (IoT) introduces several technical and managerial challenges when it comes to the use of data generated and exchanged by and between various Smart, Connected Products (SCPs) that are part of an IoT system (i.e., physical, intelligent devices with sensors and actuators). Added to the volume and the heterogeneous exchange and consumption of data, it is paramount to assure that data quality levels are maintained in every step of the data chain/lifecycle. Otherwise, the system may fail to meet its expected function. While Data Quality (DQ) is a mature field, existing solutions are highly heterogeneous. Therefore, we propose that companies, developers and vendors should align their data quality management mechanisms and artefacts with well-known best practices and standards, as for example, those provided by ISO 8000-61. This standard enables a process-approach to data quality management, overcoming the difficulties of isolated data quality activities. This paper introduces DAQUA-MASS, a methodology based on ISO 8000-61 for data quality management in sensor networks. The methodology consists of four steps according to the Plan-Do-Check-Act cycle by Deming.

## 1. Introduction

“Our economy, society and survival aren’t based on ideas or information—they’re based on things” [1]. This is one of the core foundations of the Internet-of-Things (IoT) as stated by Ashton, who coined the term. IoT is an emerging global internet-based information architecture facilitating the exchange of goods and services [2]. IoT systems are inherently built on data gathered from heterogeneous sources in which the volume, variety and velocity of data generation, exchanging and processing are dramatically increasing [3]. Furthermore, there is a certain emergence of IoT semantic-oriented vision which needs ways to represent and manipulate the vast amount of raw data expected to be generated from and exchanged between the “things” [4].

The vast amount of data in IoT environments, gathered from a global-scale deployment of smart-things, is the basis for making intelligent decisions and providing better services (e.g., smart mobility as presented in [5]). In other words, data represents the bridge that connects cyber and physical worlds. Despite of its tremendous relevance, if data are of inadequate quality, decisions from both humans and other devices are likely to be unsound [6,7]. As a consequence, Data Quality (DQ) has become one of the key aspects in IoT [6,8,9,10]. In IoT, and in particular, Smart, Connected Products (SCPs), have concrete characteristics that favour the apparition of problems due to inadequate levels of data quality. Mühlhäuser [11] defines smart, connected products (SCPs) as “entities (tangible object, software, or service) designed and made for self-organized embedding into different (smart) environments in the course of its lifecycle, providing improved simplicity and openness through improved connections”. While some of the SCP-related characteristics might be considered omnipresent (i.e., uncertain, erroneous, noisy, distributed and voluminous), other characteristics are more specific and highly dependent on the context and monitored phenomena (i.e., smooth variation, continuous, correlation, periodicity or Markovian behaviour) [6].

Also, outside of the IoT research area, DQ has been broadly studied during last years, and it has become a mature research area capturing the growing interest of the industry due to the different types of values that companies can extract from data [12]. This fact is reflected by the standardization efforts like ISO/IEC 25000 series addressing systems and software quality requirements and evaluation (SQuaRE) [13], or ISO 8000-60 series concerning the best practices in data quality management processes. In order to assure adequate levels of data quality, it is necessary to produce and implement methods, processes, and specific techniques for managing data concerns. We pose that such standards can be tailored and used within the IoT context, not only bring benefits standardizing solutions and enabling a better communication between partners. Also, the number of problems and system fails on the IoT environment is reduced, better decisions can be taken due to a better quality of data, all stakeholders are aligned and can take benefit of the advances on the standard used, and it is easier to apply data quality solutions in a global way because the heterogeneity is reduced.

Due to the youth of IoT, and despite DQ standards, frameworks, management techniques and tools proposed in the literature, DQ for IoT has not been yet widely studied. However, and prior to this research line, it is possible to cite some works that had addressed some DQ concerns in sensor wireless networks [8,14], or in data streaming [15,16] among other proposals [6]. However, these works have not considered the management of DQ in a holistic way in line with existing DQ-related standards. In our attempt to align the study of DQ in IoT to international standards, this paper provides practitioners and researchers with DAQUA-MASS, a methodology for managing data quality in SCP environments, which considers some of the DQ best practices for improving quality of data in SCP environments aligned to ISO 8000-61 [17]. Due to the intrinsic distributed nature of IoT systems, using such standards will enable the various organizations to be aligned to the same foundations, and in the end, to work in a seamless way, what will undoubtedly improve the performance of the business processes.

The remainder of this paper is organized as follows: Section 2 presents the most challenging data quality management concerns in the context of the SCP environments; Section 3 explores related work. Section 4 explains the data quality model in which our methodology is based on. Section 5 presents our proposed methodology for managing data quality in SCP environments. Finally, Section 6 discusses conclusions and implications of this work.

## 2. Data Quality Challenges in SCP Environments

This section introduces some general ideas about Smart, Connected Products (SCPs) operations, as an essential part of IoT. In addition, some challenges related to DQ in SCP environments are also introduced.

According to Cook et al. [18], a smart environment is a small world where all kinds of smart devices are continuously working to make inhabitants’ lives more comfortable. According to Mühlhäuser [11], SCP provides intelligent actions through improved connections by means of context-awareness, semantic self-description, proactive behaviour, multimodal natural interfaces, AI planning, and machine learning. 

SCPs have three main core components: physical, smart, and connectivity components. Smart components extend the capabilities and value of the physical components, while connectivity extends the capabilities and value of the smart components. This enables some smart components to exist outside the physical product itself, with a cycle of value improvement [19].

IoT and SCP can be confused in some contexts. However, IoT simply reflects the growing number of SCPs and highlights the new opportunities they can represent. IoT, which can involve people or things, is a mean for interchanging information. What makes SCPs essentially different is not the Internet, but the changing nature of the “things” [19]. A product that is smart and connected to the cloud could become part of an interconnected management solution; and companies can therefore evolve from making products to offering more complex, higher-value services within a “system of systems” [20].

SCPs include processors, sensors, software and connectivity that allow data to be exchanged between the product and its environment. The data collected by sensors of these SCPs can be then analysed to inform decision-making, enable operational efficiencies and continuously improve the performance of the product. This paper focuses on the data produced by such sensors, and how inadequate levels of data quality may affect the processing of the data, while smart and connectivity parts of SCPs are outside of the scope of this paper.

**Figure 1:**
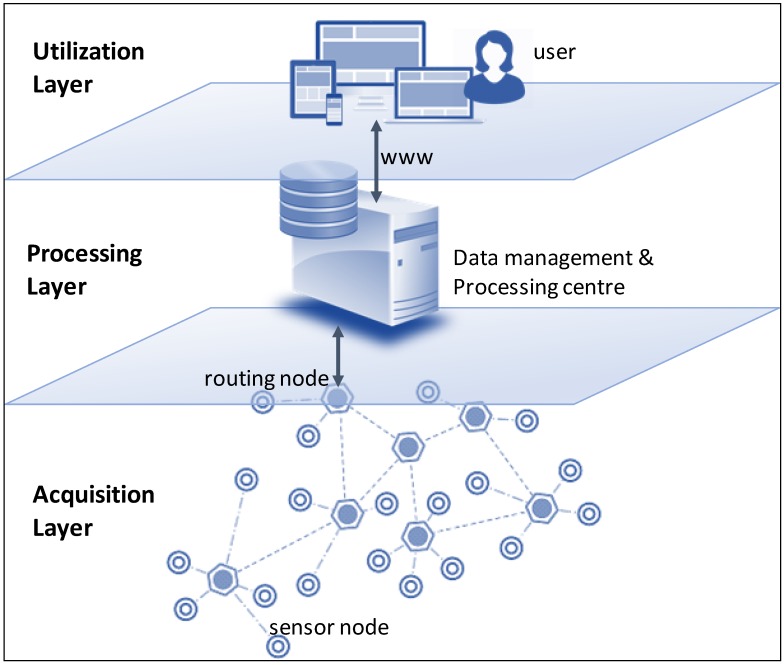
Layers in SCP environments.

SCPs can be connected in large, complex networks throughout three different layers [9]: acquisition, processing and utilization layer (see Figure 1).

- Acquisition layer refers to the sensor data collection system where sensors, raw (or sensed) and pre-processed data are managed. This is the main focus of this paper.
- Processing layer involves data resulting from data processing and management centre where energy, storage and analyse capabilities are more significant.
- Utilization layer concerns delivered data (or post-processed data) exploited, for example, over a GIS or combined with other services or applications.

As previously stated, the scope of the paper is limited to the data produced by SCPs’ sensors. Hence, the proposal is mainly intended to be applied in the context of the acquisition layer. Nevertheless, the management of the data quality in sensors can impact on how data is processed (processing layer) and how data may be used later (utilization layer).

Networking and management of SCP operations can generate the business intelligence needed to deliver smart services. Smart services are delivered to or via smart objects that feature awareness and connectivity [21]. SCP can carry out the following functions to support smart services [22]: status, diagnostics, upgrades, control and automation, profiling and behaviour tracking, replenishment and commerce, location mapping and logistics, etc.

SCP operations enable new capabilities for companies, although also arising new problems and challenges must be taken into account. On one hand, SCP operations require companies to build and support an entirely new technology infrastructure [19]. Technological layers in the new technology landscape include new product hardware, embedded software, connectivity, a product cloud running on remote servers, security tools, gateway for external information sources, and integration with enterprise business systems. On the other hand, SCP operations can provide competitive advantages, which are based on the operational effectiveness. Operation effectiveness requires to embrace best practices along the value chain, including up-to-date product technologies, the latest production equipment, and state-of-the-art sales force methods, IT solutions, and so forth. Thus, SCP operations also creates new best practices across the value chain [19].

According to the different sources of data in these SCP environments, we can distinguish different types of aggregated data:

- Sensor data: data that is generated by sensors and digitalized in a computer-readable format (for example, the camera sensor readings).
- Device data: It is integrated by sensor data; observed metadata (metadata that characterizes the sensor data, e.g., timestamp of sensor data); and device meta data (metadata that characterizes the device, e.g., device model, sensor model, manufacturer, etc.), so device data, for example, can be data coming from the camera (device).
- General data: data related to/or coming from devices which has been modified or computed to derive different data plus business data (i.e., data for business use such as operation, maintenance, service, customers, etc.).
- IoT data: general data plus device data.

A reduction in the levels of quality of these data due to different problems in SCP operations can threaten the success factors of SCP environments [6]. The quality of produced data is often affected by dysfunctional SCP devices and sensors, which are the sources providing data, and can potentially result in inadequate levels of quality that are only detected later on, when data are being processed and used. Therefore, while we can identify dysfunctional SCP devices through the analysis of sensor data by using data quality management techniques, it is noteworthy that these devices will impact the rest of the sensor network. According to [6], Table 1 summarizes some of these SCP factors that, in some cases, could condition or lead to data quality issues. In addition, the three columns on the right of Table 1 show (marked with a cross) the most critical layers affected in a greater extent by every SCP factor.

Tilak et al. [23] provide a taxonomy of sensor errors. These errors are directly related to different data quality problems in the acquisition layer. The mentioned taxonomy distinguishes the following six types of data sensors errors (see Table 2). Apart from errors in isolated SCP devices, there are other communication errors which can happen at SCP network level [23]. Table 3 summarizes the main types of communication errors: omission, crashes, delay and message corruption. The table shows the DQ issue derived by each problem, the root cause and possible solution.

All the mentioned SCP devices/sensor errors will lead to different DQ problems in the three layers depicted in Figure 1. As previously mentioned, DQ problems can be represented as a degradation of some DQ characteristics that are especially important in different environments. Let us consider two groups of data quality characteristics:

- DQ characteristics to assess data quality in use within a specific context. This aspect considers selected criteria to estimate the quality of raw sensor data at the acquisition and processing layer. There are some DQ characteristics considered, which make it possible to estimate the quality on data sources, their context of acquisition and their transmission to the data management and processing. These DQ characteristics are accuracy and completeness according to ISO/IEC 25012 [25] and reliability and communication reliability as proposed in [9]. It is also related to the utilization layer and includes availability regarding ISO/IEC 25012 [25] plus timeliness and adequacy as defined in [9].
- DQ Characteristics aimed at managing internal data quality. The main goal of managing internal data quality is to avoid inconsistent data and maintain the temporality of sensor data at the processing layer. These characteristics are consistency and currency according to ISO/IEC 25012 [25] and volatility as proposed in [9].

## 3. Related Work

The goal of this section is twofold. First, Section 3.1 presents some works related to the study of data quality in sensor networks and SCP environments in general. Second, Section 3.2 introduces and compares some data quality methodologies in order to draw the main contribution of the proposed methodology. 

### 3.1. Sensor Data Quality 

There are some published works in the literature that address the concerns related to data quality management in SCP and IoT environments. Karkouch et al. [6] presented a state-of-the-art survey for DQ in IoT. This survey presents IoT-related factors endangering the DQ and their impact on various DQ characteristics. Also, DQ problems manifestations are discussed (and their symptoms identified) as well as their impact in the context of IoT. Gonçalo et al. in [8] provided a similar survey addressing the problem of not being able to ensure desired DQ levels for dependable monitoring when using wireless sensor networks. This work pays special attention to comprehension of which faults can affect sensors, how they can affect the quality of the information and how this quality can be improved and quantified. Gutiérrez Rodríguez and Servigne in [9] also analysed data errors in sensor networks, in particular in environmental monitoring systems. In this paper, authors address the problem of uncertainty of data coming from sensors with an approach dedicated to providing environmental monitoring applications and users with data quality information. Badawy et al. [26] combined parametric and non-parametric signal processing and machine learning algorithms for automating sensor data quality control, which can identify those parts of the sensor data that are sufficiently reliable for further analysis and discards useless data.

Another research subarea of DQ in sensor networks is the DQ management in sensor data streams. Klein et al. [10] presented a quality-driven load shedding approach that screens the data stream to find and discard data items of minor quality. Thus, DQ of stream processing results is maximized under adverse conditions such as data overload. Campbell et al. [15] advocated for automated quality assurance and quality control procedures based on graphical and statistical summaries for review and track the provenance of the data in environmental sensor streams.

Other works focus on data management from different viewpoints. For example, Al-Ruithe et al. [27] detailed roles, responsibilities and policies in the context of IoT-Cloud converged environments and provide a generic framework for data governance and security. Similarly, Qin et al. [14] provided a data management perspective on large-scale sensor environments applications posing non-functional requirements to meet the underlying timeliness, reliability and accuracy needs in addition to the functional needs of data collection. Although all these approaches are interesting and provide a useful vision, they still do not address how to make available (e.g., institutionalize) best practices in data quality management to the entire organization. Such approach has been proven to be more efficient when it comes to create an organizational data quality culture. This vision is specifically important in the case of IoT, since the SCP operations can be executed across different networks belonging to different departments or even organizations. From our point of view, this is a critical aspect for IoT that must be covered in a holistic way.

### 3.2. Data Quality Methodologies Comparison

There are some methodologies that can be used as drivers for assessing and managing DQ. First, Lee et al. [28] proposed AIMQ as a methodology that encompasses a model of data quality, a questionnaire to measure DQ, and analysis techniques for interpreting the DQ measures. This methodology is mainly used to analyse the gap between an organization and best practices, as well as to assess gaps between information systems professionals and data consumers. The application of this methodology is useful for determining the best area for DQ improvement activities. This methodology has not been widely used and in a greater extent it has been considered to be too theorical and dependent on the domain. McGilvray [29] provides a practical approach for planning and managing information quality. In comparison with the methodology proposed by Lee et al., McGilvray’s one provides a more pragmatic and practical approach to achieving the desired state of DQ within an organization. However, this methodology is still dependent on the domain of application. ISO/TS 8000-150:2011 [30] “specifies fundamental principles of master data quality management, and requirements for implementation, data exchange and provenance”. This standard constitutes an informative framework that identifies processes for DQ management. This framework could be used in conjunction with, or independently of, quality management systems standards, for example, ISO 9001 [31].

Batini et al. [32] provided a literature review about different methodologies for data quality assessment and improvement. Most of the methods and techniques included in such review cannot be considered as a DQ management methodologies since do not consider all the managerial concerns in a holistic manner. At the contrary, most of these methods are focused on DQ assessment or improvement in isolation. Similar to the mentioned review, a most recent study developed by Woodall et al. [33] classified most recent DQ assessment and improvement methods. This work suffers the same problem than the work of Batini et al. Apart of these methodologies, there is a lack of comprehensive methodologies for the assessment and improvement of DQ in the domain of SCP operations and their underlaying data.

The recent standard ISO 8000-61 [17] provides a set of standard guidelines for managing DQ in a holistic way, which can be tailored for different domains. However, its main purpose is not to serve as a methodology for DQ management per se, but it simply provides a process reference model. In this sense, the standard is more descriptive than operative, what makes it not usable out-of-the-box. Aligned with this standard, this paper proposes the DAQUA-MASS methodology to deal with DQ in SCP environments. The main contribution of DAQUA-MASS methodology is that it takes the standard best practices for depicting an operative way to manage DQ (as depicted in the processes of ISO 8000-61) and tailors these to the particular domain of SCP environments, and in particular, in sensor-related data.

## 4. DAQUA-Model: A Data Quality Model

Reviewing the literature, it is possible to find that the concept of data quality has been defined in different ways. The widest used definitions are aligned with the concept of “fitness for use”. In [34], it is defined as: “Data Quality is data that is fit for use by data consumer. This means that usefulness and usability are important aspects of quality”. Different stakeholders can have different perceptions of what quality means for the same data [35]. It largely depends on the context in which data is used. Thus, DQ in IoT environments must be adequately managed considering the very nature of the IoT systems. Typically, to improve data quality, a Plan-Do-Check-Act (PDCA) cycle specifically tailored for the context of usage is followed. In this sense, we think that adopting the processes of ISO 8000-61 which are deployed in the PDCA [36] order can largely help to provide a methodology for managing data quality in IoT environments. At the core of the PDCA cycle for IoT environments is the identification of a Data Quality Model (DQModel) which, being composed of several data quality characteristics suitable for the problem, is used to identify and represent the data quality requirements required in the context. [9].

In our case, and according to our philosophy of aligning with international standards, the DQ Model proposed, is a specialization of the DQ Model introduced in ISO/IEC 25012 [25]. This DQ Model is widely accepted and used in the industry. Nevertheless, it has not been specifically developed for considering SCP aspects. In fact, the scope section of such standard literally states that it “does not include data produced by embedded devices or real time sensors that are not retained for further processing or historical purposes” [25]. Therefore, in order to complement the standard, we provide some orientation in this paper on how to specifically use ISO/IEC 25012 in the context of SCP environments.

The DQ Model focuses on the quality of the data as part of an information system and defines quality characteristics for target data used by humans and systems (i.e., the data that the organization decides to analyse and validate through the model). This model categorizes quality attributes into fifteen characteristics and considers three perspectives: inherent, system-dependent and both jointly (see crosses in Table 4).

As part of the methodology, we propose the association of the DQ characteristics with some of the sensor data errors previously shown in Table 2. These relationships suggest that erroneous data generated due to some malfunctioning sensors could lead or affect some of the DQ characteristics. We distinguish these relationships in Table 4 with ‘P’ and ‘S’ respectively signifying that a sensor error can have a primary or secondary impact in DQ characteristics. Primary means a direct (or more critical) effect while secondary is a collide (or less critical) impact.

In the following paragraphs, we introduce and summarize the fifteen DQ characteristics and, as a main contribution in this part, we provide a vision on how these DQ characteristics are tailored for SCPs operations. Along with the definitions, for the sake of the understandability an example is also provided for each characteristic. These examples are specially provided for the interpretation of these DQ characteristics in the acquisition layer, which is the scope of this paper. However, it has to be noticed that all these DQ characteristics can be considered for all the SCP layers with different ways of assessment and interpretation.

From the point of view of data quality management, it is quite important not to make the mistake of confusing the readings of dysfunctional sensors with inadequate levels of data quality, even when a dysfunctional sensor can produce data without having adequate levels of quality (i.e., not fitting the purpose of the use of data): the reason for making this distinction is that fixing errors due to dysfunctional sensors requires first fixing the sensors; on the other hand, if one can assure that the root cause is not grounded on a dysfunctional sensor, but on the data itself, then, to fix data quality errors, then data quality management techniques should be used since it should not be ignored what data means. The description of the data quality characteristics can be found in the following paragraphs:

- Accuracy. It is the degree to which data has attributes that correctly represent the true value of the intended attribute of a concept or event in a specific context of use. In SCP environments, a low degree of accuracy could be derived from devices that provide values that could differ from the value on the real world. For example, a low degree of accuracy can be when a humidity sensor reads a value of 30% and the real value is 50%. Low levels of accuracy could be directly related to sensor errors such as constant or offset, outlier errors and noise errors. Also, accuracy could be indirectly affected by continuous varying or drifting and trimming error (see Table 4).
- Completeness. It is the degree to which subject data associated with an entity has values for all expected attributes and related entity instances in a specific context of use. In SCP environments, a low degree of completeness could be derived from devices that are reading and sending no values. For example, a low degree of completeness can happen when the records of sensor data have missing values. Low levels of completeness could be related directly to sensor errors such as crash or jammed errors and indirectly, trimming and noise errors (see Table 4).
- Consistency. It represents the degree to which data has attributes that are free from contradiction and are coherent with other data in a specific context of use. It can be either or both among data regarding one entity and across similar data for comparable entities. In SCP environments, a low degree of consistency could happen when two sensors produce contradictory data. For example, for proximity sensors that provide the relative distance to the sensor position, a consistency problem for a single sensor could be a negative distance value, while a consistency problem between two sensors in the same position could be two different values. Thus, low levels of consistency could be related with continuous varying/drifting error and indirectly with constant or offset errors, trimming and noise error (see Table 4).
- Credibility. It is defined as the degree to which data has attributes that are regarded as true and believable by users in a specific context of use. In SCP environments, a low degree of credibility could be derived from a single sensor placed in someplace and the data cannot be validated by another entity or even sensor. For example, a credibility issue could happen when a sensor whose data is compared with another sensor placed near does not match. Low levels of credibility could be related directly to sensor errors such as outlier errors and indirectly with constant or offset error, continuous varying/drifting error and noise error (see Table 4).
- Currentness. It is the degree to which data has attributes that are of the right age in a specific context of use. In SCP environments, a low degree of currentness could be derived from a sensor that can be indicating past values as current value (see Table 4). For example, if an irrigation sensor produces a value that indicates that the field must be irrigated, it has been irrigated and the data is not updated. The data indicates whether it is necessary irrigation, or it is already irrigated, so this would be data without sufficient currentness.
- Accessibility. It is the degree to which data can be accessed in a specific context of use, particularly by people who need supporting technology or special configuration because of some disability. In SCP environments, a low degree of accessibility could be derived due to the necessary user is not allowed in the precise moment. For example, data produced by a specific sensor is unreachable due to network issues.
- Compliance. It refers to the degree to which data has attributes that adhere to standards, conventions or regulations in force and similar rules relating to data quality in a specific context of use. In SCP environments, a low degree of compliance could be derived from data sensor that is not being using the standards formats established on the organization. For example, if the organization establishes that for distance sensors the unit for values is meters, and if some sensors produce values expressed in meters and other in miles these data have low compliance levels.
- Confidentiality. It is the degree to which data has attributes that ensure that it is only accessible and interpretable by authorized users in a specific context of use. In SCP environments, a low degree of confidentiality could be derived from an inefficient security management of sensor data. For example, a confidentiality leak might happen when data produced by a sensor placed in a nuclear power plant can be freely accessed from external networks even when this data was marked as sensible and, therefore, confidential in order to prevent possible terrorist acts.
- Efficiency. It is the degree to which data has attributes that can be processed and provide the expected levels of performance by using the appropriate amounts and types of resources in a specific context of use. For example, a sensor send data about where is placed and send a code and a description every time the sensor sends o stores a record, it has low efficiency because only the code is enough to know all about the place. In SCP environments, a low degree of efficiency could be derived from the storage of duplicated data that could take more time and resources to send or manipulate the data.
- Precision. It is the degree to which data has attributes that are exact or that provide discrimination in a specific context of use. In SCP environments, a low degree of precision could be derived from devices that are providing inexact values as in the next example. For example, sensor data that store weight with no decimals and it is required a minimum of three decimals. Low levels of consistency could be related directly with trimming errors, and indirectly with noise errors (see Table 4).
- Traceability. The degree to which data has attributes that provide an audit trail of access to the data and of any changes made to the data in a specific context of use. In SCP environments, a low degree of traceability could be derived from sensor data with no metadata. For example, data logs contain information about who has acceded to sensor data and operations made with them. Low levels of traceability could be related indirectly to crash or jammed errors as well as to temporal delay errors (see Table 4).
- Understandability. The degree to which data has attributes that enable it to be read and interpreted by users, and are expressed in appropriate languages, symbols and units in a specific context of use. In SCP environments, a low degree of understandability could be derived from sensor data represented with codes instead of acronyms. For example, records of data about temperature on a car has an attribute to know the place of the sensor in the car. This attribute can be stored as a code like “xkq1”, but if is stored as “GasolineTank” it is supposed to have a higher level of understandability.
- Availability. The degree to which data has attributes that enable it to be retrieved by authorized users and/or applications in a specific context of use. In SCP environments, a low degree of availability could be derived from the insufficient resources of the system in which sensor data is stored. For example, to assure sensor data availability, sensor replication can be used to make it available even if there is some issue on a sensor. Low levels of availability could be related indirectly with temporal delay error and crash or jammed errors (see Table 4).
- Portability. The degree to which data has attributes that enable it to be installed, replaced or moved from one system to another preserving the existing quality in a specific context of use. For example, sensor data is going to be shared with a concrete system or even other organization or department, data loss can occur. If this happens, for example, due to a data model mismatching or a problem with the data format, the reason is directly related to portability of data. In SCP environments, a low degree of portability could be derived from sensor data that does not follow a specific data model (see Table 4) or the format present some problems.
- Recoverability. The degree to which data has attributes that enable it to maintain and preserve a specified level of operations and quality, even in the event of failure, in a specific context of use. In SCP environments, a low degree of recoverability could be derived from devices that does not have a mechanism failure tolerant or backup. For example, when a device has a failure, data stored in that device should be recoverable. Low levels of recoverability could be related indirectly with temporal delay error and crash or jammed errors (see Table 4).

Although our DQ Model considers initially all the DQ characteristics defined in ISO/IEC 25012, it could be necessary to customize the DQ characteristics chosen to adapt them into the specific SCP context. This customization might depend on the concrete organization and how it applies the methodology to specific SCP contexts. The customized model will conform the data quality model for an organization with a specific SCP environment.

## 5. DAQUA-MASS: A Data Quality Management Methodology for Data Sensors

This section presents DAQUA-MASS, an ISO 8000-61-based Data Quality Management methodology for data sensors. Steps provided in this methodology are based in some of the processes introduced by ISO 8000-61 [17], which, as we previously said, gathers the best practices around data quality management by means of a process approach. Each process in this standard is described by means of a purpose, the outcomes and the activities that are to be applied for the assurance of data quality. Thus, this standard mainly covers: (1) fundamental principles of data quality management; (2) the structure of the data quality management process; (3) definitions of the lower level processes for data quality management; (4) the relationship between data quality management and data governance; (5) implementation requirements. This standard is used along with ISO 8000-62 to assess and improve the organizational maturity when it comes to data quality management. However, in this paper, we consider the processes in ISO 8000-61 to rigorously depict DAQUA-MASS.

Steps of DAQUA-MASS are grouped in four phases according to the Plan-Do-Check-Act cycle, as it is done in 8000-61. PDCA is implicitly a closed loop, signifying that the process is iterative and last phase of every iteration provides feedback on starting a new iteration. Many acknowledged models, such as the in IoT information fusion [37] or the JDL model [38], are all closed loops. By this way, they can be self-improved to adjust the dynamic world to maximize the performance. The IoT is a dynamic system so, the data quality management is adapted to the changes in every loop of the methodology. Each loop serves to adapt to changes and new quality needs that may arise. The methodology is designed so that the complete cycle is iteratively executed depending on the goals, needs and resources of the organization. The PDCA cycle will contribute to more effective and efficient data quality and consists of seven steps grouped in the following four phases:

- The plan phase establishes the strategy and the data quality improvement implementation plan as necessary to deliver results in accordance with data requirements;
- In the do phase the data quality improvement implementation plan is executed;
- During the check phase, it is conducted the monitorization of data quality and process performance against the strategy and data requirements and report the results to validate the efficiency of the corrective actions; and finally
- The act phase takes actions to continually improve process performance.

**Figure 2:**
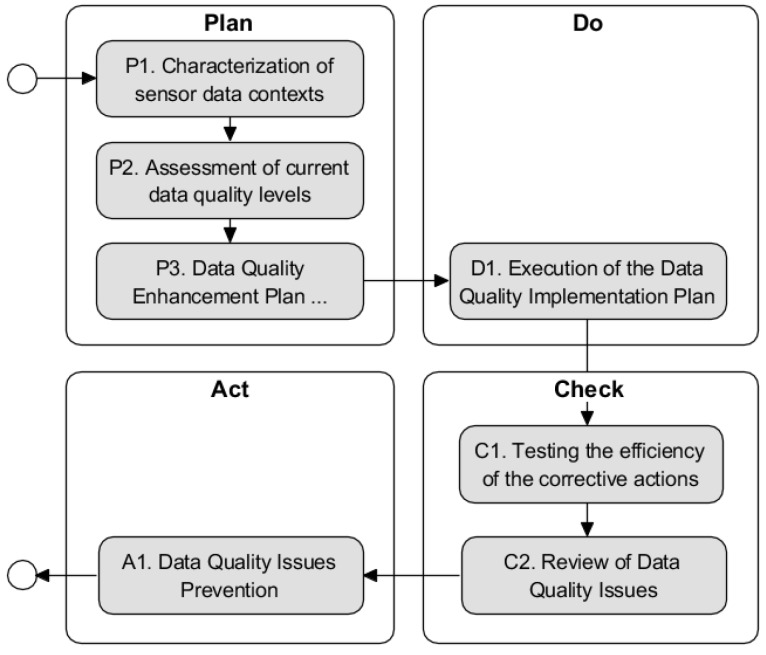
Methodology DAQUA-MASS phases and steps.

Figure 2 summarizes the step flow throughout the four phases. Every step is defined with a general description and set of concrete activities. Following sections present all the methodology steps grouped in the mentioned Plan-Do-Check-Act cycle. The definition for all the steps provides a table depicting all the expected input and output generated by each activity in the step as well as a RACIQ matrix (R—Responsible (works on); A—Accountable, C—Consulted; I—Informed; Q—Quality Reviewer) which indicates the role of stakeholders involved in the activity.

We consider the following list of stakeholders to be involved in the application of this methodology. It should be notice that there are alternative, similar (even overlapped) role definitions in the context of data quality management and SCP environments. However, we consider these are the most common and well-known stakeholders, and other that are not mentioned can be easily assimilated to one of the proposed.

- Chief Information Officer (CIO). It is the most senior executive in an enterprise responsible for the traditional information technology and computer systems that support enterprise goal.
- Chief Data Officer (CDO). It is a corporate officer responsible for enterprise wide data governance and utilization of data as an asset, via data processing, analysis, data mining, information trading and other means. CIO and CDO are at the executive level.
- Data Governance Manager (DGM). He or she is overseeing enterprise data governance program development and is responsible for architecting certain solutions and frameworks. It is at the strategic level.
- Data Quality Steward for SCP domain (SCP DQ Steward). It is a DQ steward at tactical level for the area of SCP environments by considering its implications in DQ.
- Data Quality Steward (DQ Steward). It is responsible for utilizing an organization’s data quality governance processes to ensure fitness of data elements—both the content and metadata. Data stewards have a specialist role that incorporates processes, policies, guidelines and responsibilities for administering organizations’ entire data in compliance with DQ policies and procedures. This is at operational level.
- SCP Technical Architect (SCP Arch). A SCP architect is a SCP (and in general IoT) expert who makes high-level design choices and dictates technical standards, including SCP technology standards, tools, and platforms.

### 5.1. The Plan Phase

The data quality planning phase establishes data requirements and objectives for data quality, creating plans to achieve the objectives and evaluating the performance of the plans. These plans balance current data quality levels, cost, resources and capabilities across the organization for the assessment of data quality of sensor data. This phase is initiated based on needs and expectations of stakeholders or the feedback of the process improvements performed during data quality improvement (the act phase). The expected result of the execution of this phase is the complete planification for adequately collect sensor data. This includes requirements management, definition of policies for data, devices lifecycle management, and it is important to highlight the definition of policies to retain the original unmanipulated data and all versions of the input data. Table 5 summarizes inputs, outputs and responsibility distribution for this phase. Following, a description of the steps grounded on the corresponding ISO 8000-61 process is introduced. At this point it is reminded that the scope of the methodology is on sensor data (see Figure 1).

#### 5.1.1. P1. Characterization of the Current State of the Sensor Data Contexts

This first step is devoted to characterizing the current state of the SCP environments and all the specific sensor data contexts. This step has the following activities:

- P1-1. Sensor and sensor data lifecycle management specification. The lifecycle of sensors [2] and sensor data [39] have to be defined so that it can be managed. For sensors, the lifecycle starts when the device is obtained, and the last step is when it is necessary to replace o remove the sensor because it became useless. For sensor data, the lifecycle starts when sensors produce the data and ends when the data is eliminated. The end of the sensor data life cycle should not be confused with the moment in which the data goes from being operational to historical data. Operational sensor data is used on a day-to-day basis, but when data is no longer useful on a day-to-day basis due to its age, these data is separated and stored in another database and become into historical data. It is important to highlight that sensor and sensor data lifecycle is used to better contextualize the environment of the data.
- P1-2. Management of quality policies, standards and procedures for data quality management. Specify fundamental intentions and rules for data quality management. Ensure the data quality policies, standards and procedures are appropriate for the data quality strategy, comply with data requirements and establish the foundation for continual improvement of the effectiveness and efficiency of data quality management in all the key SCP operations. The purpose of data quality policy/standards/procedures management is to capture rules that apply to performing data quality control, data quality assurance, data quality improvement, data-related support and resource provision consistently. Before the implementation and definition of the complete plan, it should be defined the policies, standards and procedures in order to define the implementation plan based on them.
- P1-3. Provision of sensor data and work specifications. Developing specifications that describe characteristics of data and work instructions for smart connected products enables Data processing and data quality monitoring and control. To support the description of the provision of the sensor data work specifications some metadata must be provided. Metadata can help with one of the biggest problems of SCP: interoperability. Interoperability refers to the ability for one or more smart connected products to communicate and exchange [15].
- P1-4. Identification, prioritization and validation of sensor data requirements. Collect the needs and expectations related to sensor data from devices and stakeholders. Then, it is translated by identification, prioritization and validation of data requirements. The purpose of Requirements Management is to establish the basis for creating or for refining a data quality strategy for SCP environments aligned to the needs and expectations of stakeholders. It is important to have well defined and implemented good sensor data requirements to avoid problems since the start and to facilitate the collection and integration of sensor data.

#### 5.1.2. P2. Assessment of the Current State of the Levels of Data Quality

This step is aimed to evaluate the current state of the data quality levels in all the surrounding SCP environments. This step has the following activities:

- P2-1. Identification of the data quality characteristics representing quality requirements and determination and development of metrics and measurement methods. Develop or select the measurement indicators, corresponding metrics and measurement methods used to measure the quality levels of data produced by devices during all the SCP operations.
- P2-2. Measurement and analysis of data quality levels. Measure the data quality levels by implementing the measurement plans and determining the measurement results. This means the application of the measurement algorithms defined and developed on the previous step (P2-1). Such algorithms strongly depend on the IT infrastructure landscape of every organization. Thereby, every organization often develop their own algorithms or use commercial solutions which are compliant with their own infrastructure. Merino et al. [7] presents algorithms and metrics for each data quality characteristic. After data quality levels have been measured, these can be quantitatively analysed to extract insights about the SCP environment being managed. As a result, a list of nonconformities can be elaborated in the next step, with which to make informed decisions.

#### 5.1.3. P3. Data Quality Enhancement Plan Definition 

Data Quality implementation planning identifies the resources and sequencing by which to perform DQ control, DQ Assurance, DQ Improvement, data-related support and resource provision across the organization. At this point, after the collection of sensor data requirements and the definition of standards and policies, it is important to configure the devices to implement the plan and place each node following a defined strategy. Optimal node placement allows to obtain the optimal value through data. This step considers the following activities:

- P3-1. Analysis of root causes of data nonconformities. Analysing the root causes of each data quality issue and assess the effect of the issue on business processes. The purpose of root cause analysis and solution development of non-solved data quality non-conformities is to establish, in accordance with the data quality strategy and with the priorities identified by Data Quality Assurance, the basis on which to perform data cleansing and/or process improvement for data nonconformity prevention.
- P3-2. Data quality risk assessment. Identify risks throughout the data life cycle, analyse the impact if each risk was to occur and determine risk priorities to establish the basis for monitoring and control of processes and data. The purpose of data quality monitoring and control is, by following applicable work instructions, to identify and respond when Data Processing fails to deliver data that meet the requirements in the corresponding data specification. It allows to control what is happening on data or even on the SCP environment. For example, with the control of sensor data quality, it is possible to identify certain issues on devices. Data is often the most important thing to protect, because although the direct cost of losing it may be small compared with research data or intellectual property. If a sensor is not functioning any more due to some reason, it can be replaced, but if a data loss is produced is very difficult to recover it or even impossible to recover. This can bring not only a data loss but can also be the root cause of other problems. In conclusion, data quality risks should be identified in order to avoid them. As Karkouch et al. highlight in [6], the main factors affecting DQ in SCP or IoT environments are: deployment scale, resources constraints, network, sensors (as physical devices), environment, vandalism, fail-dirty, privacy preservation, security vulnerability and data stream processing.
- P3-3. Development of improvement solutions to eliminate the root causes. Propose solutions to eliminate the root causes and prevent recurrence of nonconformities. Evaluate the feasibility of the proposed improvements through cost benefit analysis.
- P3-4. Definition of improvement targets. The purpose of this activity is to analyse possible improvements areas according to the business processes, risk catalogue and the data quality strategy; and then it selects those that are more aligned with the data quality strategy and/or are able to lead to greater data quality enhancement regarding previous detected risks. Specific areas or sub-nets of devices in the organization’s SCP environments could also serve as a criterion to determine specific improvement targets.
- P3-5. Establishment of the data quality enhancement plan. Define the scope and target of data quality and prepare detailed implementation plans, defining and allocating the resources needed.

### 5.2. The Do Phase

This phase consists of the data quality control that is carried out based on the implementation plan established in data quality planning (see the Plan phase). The phase, when completed, delivers data that meet the specified requirements. This phase involves creating, using and updating data according to specified work instructions and monitoring quality by checking whether the data conform to predetermined specifications.

In this phase, it is important to address aspects such as the provision of complete metadata, the use of flags to convey information about data, the documentation of all sensor data processing, monitoring and control of data, maintain appropriate levels of human inspection, perform range, domain and slope checks, implement an automated alert system for sensor network issues or automate sensor data quality procedures. Table 6 summarizes inputs, outputs and responsibility distribution for this phase.

#### D1. Execution of the Data Quality Improvement Plan

The purpose of Provision of Data Specifications and Work Instructions is to establish the basis on which to perform Data Processing and Data Quality Monitoring and Control, taking account of the outcomes of the Data Quality Planning, the Data-Related Support and the Resource Provision processes. Having the plan obtained for the first phase, the first step is to provide data and work specification to collect data from devices on the defined way. This step considers the following activities:

- D1-1. Establish flags to convey information about the sensor data. Flags or qualifiers convey information about individual data values, typically using codes that are stored in a separate field to correspond with each value. Flags can be highly specific to individual studies and data sets or standardized across all data.
- D1-2. Definition of the optimal node placement plan. It is a very challenging problem that has been proven to be NP-hard (non-deterministic polynomial-time hardness) for most of the formulations of sensor deployment [40,41]. To tackle such complexity, several heuristics have been proposed to find sub-optimal solutions [42,43]. However, the context of these optimization strategies is mainly static in the sense that assessing the quality of candidate positions is based on a structural quality metric such as distance, network connectivity and/or basing the analysis on a fixed topology. Also, application-level interest can vary over time and the available network resources may change as new nodes join the network, or as existing nodes run out of energy [44]. Also, if we talk about node placement, node or sensor replication. Replicating data sensors is important for purposes of high availability and disaster recovery. Also, replication of this data on cloud storage needs to be implemented efficiently. Archiving is one way to recover lost or damaged data in primary storage space, but replicas of data repositories that are updated concurrently with the primary repositories can be used for sensitive systems with strong data availability requirements. Replication can be demanding in terms of storage and may degrade performance due to if a concurrent updates strategy is enforced.
- D1-3. Redesign the software or hardware that includes sensors to eliminate root causes. It is an alternative process to the optimal re-placements of sensors. For example, redesign and reimplement a fragment of a SCP’s firmware could improve fault tolerance.
- D1-4. Data Cleansing. The purpose of Data Cleansing is to ensure, in response to the results of root cause analysis and solution development, the organization can access data sets that contain no nonconformities capable of causing unacceptable disruption to the effectiveness and efficiency of decision making using those data. Also, the nonconformities are corrected implementing developed solutions and make a record of the corrections.
- D1-5. Force an appropriate level of human inspection. If performed by trained and experienced technicians, visual inspection is used to monitor the state of industrial equipment and to identify necessary repairs. There are also technologies used to assist with fault recognition or even to automate inspections. Shop floor workers are made responsible for basic maintenance including cleaning machines, visual inspection and initial detection of machine degradation.
- D1-6. Implement an automated alert system to warn about potential sensor data quality issues. Having only human inspection can be a complex task for maintenance staff. It is necessary to implement a system in which some key indicators are constantly reading if the sensors status is correct or not. If a sensor is not working properly, the root cause can be due to a hardware, network or software failure and affects to data quality. Furthermore, an automated data quality procedure might identify anomalous spikes in the data and flag them. Even though, it is almost always necessary the human supervision, intervention and inspection as stated in [45,46]; the inclusion of automated quality is often an improvement, because it ensures consistency and reduces human bias. Automated data quality procedures are also more efficient at handling the vast quantities of data that are being generated by streaming sensor networks and reduces the amount of human inspection required.
- D1-7. Schedule sensor maintenance to minimize data quality issues. Sensors require routine maintenance and scheduled calibration that, in some cases, can be done only by the manufacturer. Ideally, maintenance and repairs are scheduled to minimize data loss (e.g., snow-depth sensors repaired during the summer) or staggered in such a way that data from a nearby sensor can be used to fill gaps. In cases in which unscheduled maintenance is required, stocking replacement parts on site ensures that any part of the network can be replaced immediately.

### 5.3. The Check Phase

The check phase consists of the data quality assurance, which measures data quality levels and the methodology steps performance related to data nonconformities or other issues that have arisen as a result of the data quality planning (see the Plan phase) or the data quality control (see the Do phase). This measurement provides evidence by which to evaluate the impact of any identified poor levels of data quality on the effectiveness and efficiency of business processes. On this phase is important to address aspects like schedule sensor maintenance and repairs to minimize data loss, measurement and evaluation of sensor data quality. Table 7 summarizes inputs, outputs and responsibility distribution for this phase.

#### 5.3.1. C1. Testing the Efficiency of the Corrective Actions

The purpose of Measurement of Data Quality is the Provision of Measurement Criteria, to generate input for the Evaluation of Measurement Results. This step considers the following activities:

- C1-1. Monitoring and control of the enhanced data. According to the identified risk priorities, monitor and measure conformity of data to the applicable specification. Monitoring and measuring takes place either at intervals or continuously and in accordance with applicable work instructions. This work instructions can be: perform range checks on numerical data, perform domain checks on categorical data and perform slope and persistence checks on continuous data streams. If data nonconformities are found, then correct the data when viable and distribute to stakeholders a record of the viability and degree of success for each corrective action.
- C1-2. Definition of an interstice comparison plan. Create policies for comparing data with data from related sensors. If no replicate sensors exist, interstice comparisons are useful, whereby data from one location are compared with data from nearby identical sensors.

#### 5.3.2. C2. Review of Data Quality Issues

The purpose of Review of Data Quality Issues is to identify the starting point for deciding to measure data quality levels and process performance with the potential to generate opportunities to improve data quality. The results of measurement and evaluation of data quality are analysed and possible issues are identified. This step considers the following activities:

- C2-1. Issue analysis. Review non-solved nonconformities arising from Data Processing to identify those that are possibly connected to the reported issue that has triggered the need for Data Quality Assurance. This review creates a set of related nonconformities. This set is the basis for further investigation through the measurement of data quality levels in SCP environments. Respond to the reporting of unresolved data nonconformities from within Data Quality Control, indications of the recurrence of types of nonconformity or other issues raised against the results of Data Quality Planning or Data Quality Control.

### 5.4. The Act Phase

The Act phase consists of the data quality improvement that involves analysing the root causes of data quality issues based on the assessment results derived from data quality assurance (see the Check phase). To prevent future data nonconformities, the steps in this phase try to correct all the existing nonconformities and also transforms SCP operations as appropriate. On this phase, it is important to address aspects as analysis of root causes of sensor data problems, management of sensor data problems, correction and prevention, make available ready access to replacement parts and anticipate common repairs and maintain inventory replacement parts. Table 8 summarizes inputs, outputs and responsibility distribution for this phase.

#### A1. Data Quality Issues Prevention

The purpose of Data Quality issues prevention results of Root Cause Analysis and Solution Development is to increase the extent to which the organization achieves a systematic and systemic approach to achieving data quality. This step tries to prevent issues on devices or, for example, to avoid data loss when it can be predicted the repair or the maintenance of devices of the SCP environment. In order to prevent the recurrence of each actual or the occurrence of each potential data nonconformity or similar data nonconformities by refining and applying guidelines, rules and procedures. To achieve this, the following activities should be conducted:

- A1-1. Make available ready access to replacement parts. Schedule routine calibration of instruments and sensors based on manufacturer specifications. Maintaining additional calibrated sensors of the same make/model can allow immediate replacement of sensors removed for calibration to avoid data loss. Otherwise, sensor calibrations can be scheduled at non-critical times or staggered such that a nearby sensor can be used as a proxy to fill gaps.
- A1-2. Update the strategy for node replacement. Controlled replacement is often pursued for only a selected subset of the employed nodes with the goal of structuring the network topology in a way that achieves the desired application requirements. In addition to coverage, the nodes’ positions affect numerous network performance metrics such as energy consumption, delay and throughput. For example, large distances between nodes weaken the communication links, lower the throughput and increase energy consumption. Additionally, it can anticipate some common repairs and maintain inventory replacement parts. This means that sensors could be replaced before failure where sensor lifetimes are known or can be estimated.

## 6. Conclusions

In this paper, we have tackled the challenges of data quality problems in SCP environments. Many research and standardization efforts have been made in the DQ area over the last years, and some interesting results have been already transferred to IoT as well. However, the approaches presented in the literature have two main drawbacks. On the one hand, these proposals do not take into account the nature of SCP environments and the concrete factors that affect the way in which DQ must be treated in such context. On the other hand, these approaches do not consider existing DQ management standards that have not been tailored yet for IoT and, more specifically, to SCP contexts. Given the importance of institutionalizing best practices in SCP for DQ management, we consider it of paramount importance to provide practitioners and organizations with techniques aligned with standards, reducing their adaptation efforts and favouring systematic and holistic approaches to the problem. As a main contribution, we have provided in this paper a Data Quality Management Methodology for Sensor Data, named DAQUA-MASS, based on ISO 8000-61. The methodology is structured according to the PDCA Cycle of continuous improvement. The methodology is composed by 7 steps divided in several activities. Input and output products are already identified for each activity in the methodology. It is noteworthy to highlight the identification of the various roles involved in the management of data quality in sensor data.

So, the data quality model along with the methodology offer a unique framework to enable designers of IoT projects including sensor networks and practitioners in charge of exploiting IoT systems to assure that the business processes working over these systems can manage data with adequate levels of quality. Once discarded data quality problems or identified data quality problems root causes, it will be easier to focus the attention exclusively on sensor networks of IoT systems. Working aligned to international open standards will enable organizations to speak the same language and devote the required efforts only to the proper working of the IoT systems, preventing to be aware of some other data quality concerns.

Future research lines are planned to be primarily focused on the empirical validation of this methodology. It will be used by different organization in various SCP environments in order to ensure its applicability in large scale. The mentioned case studies will allow us to provide different specializations of the data quality model (or recommendation on how to tailor it) to different organization depending on the domain. In parallel, other future research lines are in accordance with some limitations of the scope considered in this paper. Thus, it is expected to be considered data quality management issues and its implications in the processing and utilization layers apart from the acquisition layer (see Figure 1). While simplified data analysis is being performed at the devices in the acquisition layer to control the device, most of analysis, diagnosis and improvement of sensor data are being performed in the processing layer. This will force to manage sensor data together with general-purpose data to achieve a holistic data quality management in the processing layer.

## Figures and Tables

**Table 1:** SCP factors that can finally affect the levels of DQ according to [6].

SCP Factor	Side Effect in Data Quality	Acquisition	Processing	Utilization
Deployment Scale	SCPs are expected to be deployed on a global scale. This leads to a huge heterogeneity in data sources (not only computers but also daily objects). Also, the huge number of devices accumulates the chance of error occurrence.	X	X	
Resources constraints	For example, computational and storage capabilities that do not allow complex operations due, in turn, to the battery-power constraints among others.	X	X	
Network	Intermittent loss of connection in the IoT is recurrent. Things are only capable of transmitting small-sized messages due to their scarce resources.		X	X
Sensors	Embedded sensors may lack precision or suffer from loss of calibration or even low accuracy. Faulty sensors may also result in inconsistencies in data sensing.	X		
Environment	SCP devices will not be deployed only in tolerant and less aggressive environments. To monitor some phenomenon, sensors may be deployed in environments with extreme conditions. Data errors emerge when the sensor experiences the surrounding environment influences [23].	X		X
Vandalism	Things are generally defenceless from outside physical threats (both from humans and animals).	X		X
Fail-dirty.	A sensor node fails, but it keeps up reporting readings which are erroneous. It is a common problem for SCP networks and an important source of outlier readings.	X	X	
Privacy	Privacy preservation processing, thus DQ could be intentionally reduced.			X
Security vulnerability	Sensor devices are vulnerable to attack, e.g., it is possible for a malicious entity to alter data in an SCP device.	X		X
Data stream processing	Data gathered by smart things are sent in the form of streams to the back-end pervasive applications which make use of them. Some stream processing operators could affect quality of the underlying data [10].Other important factors are data granularity and variety [24]. Granularity concerns interpolation and spatio-temporal density while variety refers to interoperability and dynamic semantics.	X	X	

**Table 2:** Sensors errors deriving DQ problems in SCP environments (adapted from [8]).

Error	Description	Example
Temporal delay error	The observations are continuously produced with a constant temporal deviation	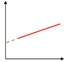
Constant or offset error	The observations continuously deviate from the expected value by a constant offset.	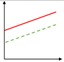
Continuous varying or drifting error	The deviation between the observations and the expected value is continuously changing according to some continuous time-dependent function (linear or non-linear).	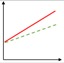
Crash or jammed error	The sensor stops providing any readings on its interface or gets jammed and stuck in some incorrect value.	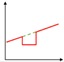
Trimming error	Data is correct for values within some interval but are modified for values outside the interval. Beyond the interval, the data can be trimmed or may vary proportionally.	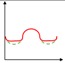
Outliers error	The observations occasionally deviate from the expected value, at random points in the time domain.	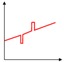
Noise error	The observations deviate from the expected value stochastically in the value domain and permanently in the temporal domain.	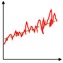

**Table 3:** SCP Network Errors.

Sensor Fault	DQ Problem	Root Cause	Solution
Omission faults	Absence of values	Missing sensor	Network reliability, retransmission
Crash faults (fading/intermittent)	Inaccuracy/absence of values	Environment interference	Redundancy/estimating with past values
Delay faults	Inaccuracy	Time domain	Timeline solutions
Message corruption	Integrity	Communication	Integrity validation

**Table 4:** DQ Characteristics in ISO/IEC 25012 that can be affected by sensor data errors.

DQ Characteristics	Inherent	System Dependent	Temporal Delay Error	Constant or Offset Error	Continuous Varying/Drifting Error	Crash or Jammed Error	Trimming Error	Outliers Error	Noise Error
			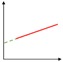	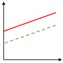	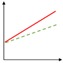	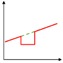	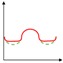	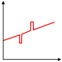	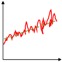
Accuracy	x			P	S		S	P	P
Completeness	x					P	S		S
Consistency	x			S	P		S		S
Credibility	x			S	S			P	S
Currentness	x		P					S	
Accessibility	x	x							
Compliance	x	x							
Confidentiality	x	x							
Efficiency	x	x							
Precision	x	x					P		S
Traceability	x	x	S			S			
Understandability	x	x							
Availability		x	S			S			
Portability		x							
Recoverability		x	S			S			

**Table 5:** Inputs, outputs and RACIQ matrix for the Plan phase. (R—Responsible (works on); A—Accountable, C—Consulted; I—Informed; Q—Quality Reviewer).

Step	Act.	Input	Output	CIO	CDO	DGM	SCP DQ Steward	DQ Steward	SCP Arch
P1	P1-1	List of prioritized data quality requirements Perform analysis definition	Specification of data lifecycle in SCP environments			I	A	C	R
	P1-2	Specification of data lifecycle in SCP environments	Data Quality Policies for SCPs Data Quality Procedures for SCPs		I	A	R	R	C
	P1-3	Specification of data lifecycle in SCP environments Data Quality Policies for SCPs Data Quality Procedures for SCPs	Meta-data specification			I	C	A R	C
	P1-4	Specification of data lifecycle in SCP environments Stakeholders or any other experts opinion	Data Quality Strategy List of prioritized data quality requirements	I	A	R	R	R	C
P2	P2-1	List of prioritized data quality requirements Mechanisms for data monitoring	Metrics list Measurement plan		I	A	C	R	
	P2-2	Metric list Measurement plan List of prioritized data quality requirements	Implementation of measurement methods			A	Q	Q	R
P3	P3-1	DQ Issues list Data Quality Strategy List of prioritized data quality requirements Business process definition	Root causes for DQ issues related with SCP faults Report with the effects of DQ issues on business processes	I	A	R	R	R Q	
	P3-2	Data Quality Strategy List of prioritized data quality requirements Business process definition	DQ risk catalogue	I	I	A	R	R	C
	P3-3	Root causes for DQ issues related with SCP faults Report with the effects of DQ issues on business processes Specification of data lifecycle in SCP environments SCP node placement plan SCP node replication plan	Solution definition for mitigating root causes		I	A	R	R	R C
	P3-4	Data Quality Strategy Business process definition DQ risk catalogue Specification of data lifecycle in SCP environments	Improvement target list						
	P3-5	Data Quality Strategy List of prioritized data quality requirements Report with the effects of DQ issues on business processes DQ risk catalogue	Data quality enhancement plan	I	Q	A	R	R	C

**Table 6:** Inputs, outputs and RACIQ matrix for the Do phase. (R—Responsible (works on); A—Accountable, C—Consulted; I—Informed; Q—Quality Reviewer).

Step	Act.	Input	Output	CIO	CDO	DGM	SCP DQ Steward	DQ Steward	SCP Arch
D1	D1-1	Meta-data specification Specification of data lifecycle in SCP environments	Sensor flags specification				A	Q	R
	D1-2	Specification of data lifecycle in SCP environments	SCP node placement plan update SCP node replication plan update			I	A	Q	R
		Root causes for DQ issues related with SCP faults Specification of data lifecycle in SCP environments	SCP hardware updates SCP software updates			I	A	Q	R
	D1-3	Root causes for DQ issues related with SCP faults Report with the effects of DQ issues on business processes SCP node placement plan SCP node replication plan	Implementation of data cleansing mechanisms			I	A Q	R	C
	D1-4	Data quality enhancement plan DQ risk catalogue Mechanisms for data monitoring	Human inspection plan		I	A	Q	R	C
	D1-5		Automated alert system				I	C Q	A R
	D1-6	Root causes for DQ issues related with SCP faults Report with the effects of DQ issues on business processes Specification of data lifecycle in SCP environments SCP node placement plan SCP node replication plan	Sensor maintenance plan			I	A	Q	R

**Table 7:** Inputs, outputs and RACIQ matrix for the Check phase. (R—Responsible (works on); A—Accountable, C—Consulted; I—Informed; Q—Quality Reviewer).

Step	Act.	Input	Output	CIO	CDO	DGM	SCP DQ Steward	DQ Steward	SCP Arch
C1	C1-1	Data quality enhancement plan List of prioritized data quality requirements DQ risk catalogue	Mechanisms for data monitoring update DQ control mechanisms update		I	A	R	R	R
	C1-2	Specification of data lifecycle in SCP environments SCP node placement and replication plan	Interstice comparison plan			I	A	Q	R
C2	C2-1	Data Quality Strategy DQ risk catalogue Implementation of measurement methods	DQ Issues list	I	I	A	R	Q	

**Table 8:** Inputs, outputs and RACIQ matrix for the Act phase. (R—Responsible (works on); A—Accountable, C—Consulted; I—Informed; Q—Quality Reviewer).

Step	Act.	Input	Output	CIO	CDO	DGM	SCP DQ Steward	DQ Steward	SCP Arch
A1	A1-1	Sensor maintenance plan SCP node placement plan SCP node replication plan Root causes for DQ issues related with SCP faults Report with the effects of DQ issues on business processes Specification of data lifecycle in SCP environments	Sensor calibration plan Replacement ready access strategy Sensor maintenance plan			I	A	C	R
	A1-2		Device replacement plan			I	A	C	R
